# Supplementary material for: Designing a Needs-Oriented Psychological Intervention for Chinese Women Undergoing an Abortion
Source: Int J Environ Res Public Health. 2022 Dec 31;20(1):782. doi: 10.3390/ijerph20010782 (PMC9819149; doi:10.3390/ijerph20010782)
Supplement: Supplementary file 1 [file ijerph-20-00782-s001.zip › ijerph-2119436-supplementary.pdf]

Supplementary File S1

The original interview guide - Chinese translation

Semi-Structured Interview questions

(Abortion patients; before discharge)

1. How would you appraise yourself? How would you appraise your family environment? School environment? Community environment?

2. What do you think about sex, premarital sex, premarital pregnancy and abortion before and post this abortion experience?

3. What do you think of contraception before and post this abortion experience? What factors do you think will affect your contraception use?

4. How do your family, peers, school, and community view sex, premarital sex, premarital pregnancy and abortion?

5. Do you think you know anything about sex or contraception? Where do you get this information? Where do you get contraceptives?

6. Could you tell me about your experience of this pregnancy and abortion experience? From doing pregnancy test up to now.

7. What do you think this pregnancy and abortion experience will influence you? How? By what extent?

8. Could you tell me what abortion care services do you receive from the clinic to help you manage the abortion? What do you think of these services? Why?

9. Could you tell me who provided the services? (E.g. nurses, physicians)? Are you satisfied with the timing/the delivery method of the services? Why/Why not?

10. How would you prefer the services to be delivered (i.e. e-book, videos, booklets, website)? Do you have a preference for whom to provide the services? At what time? Why?

11. Will you use contraception when having sex in the future? What kind of contraception should you choose? Why?

12. What do you think would be your chances of a repeat unintended pregnancy? Will you choose to have an abortion next time? why?

Supplementary File S2

Assessment list for data fitness

**Categories**	**Items**
Review and assessment of provenance	
	Where did it come from?
	How were data constructed
	What was the relationship of the researcher to the participants?
	Quality of transcription
Available information from various level of context	
	Level of the study: interview transcripts with detailed annotation
	Level of fieldwork interaction: information about the fieldwork setting, and detail about research planning and fieldwork implementation
	Macro-level: Information about the broader social, cultural or economic context within which the research takes place

Supplementary File S3

Data analysis matrices

Process / Matrices	Relevant content	Grouping and categorization	Abstraction
**First appointment**			
Specific stressor			
Needs			
Support preferences			
**During abortion**			
Specific stressor			
Needs			
Support preferences			
**Post-abortion**			
Specific stressor			
Needs			
Support preferences			
